# Using parental phenotypes in case-parent studies

**DOI:** 10.3389/fgene.2015.00221

**Published:** 2015-06-23

**Authors:** Min Shi, David M. Umbach, Clarice R. Weinberg

**Affiliations:** Biostatistics and Computational Biology Branch, National Institute of Environmental Health SciencesResearch Triangle Park, NC, USA

**Keywords:** case-parent triad, parental phenotype, association study, SNPs, likelihood ratio test

## Abstract

In studies of case-parent triads, information is often collected about history of the condition in the parents, but typically parental phenotypes are ignored. Including that information in analyses may increase power to detect genetic association for autosomal variants. Our proposed approach uses parental phenotypes to assess association independently of the usual case-parent-based association test, enabling cross-generational internal replication for findings based on offspring and their parents. Our model for parental phenotypes also resists bias due to population stratification. We combine the information from the two generations into a single coherent model that can exploit approximate equality of parental and offspring relative risks to improve power and can also test that equality. We call the resulting procedure the Parent-phenotype Informed Likelihood Ratio Test (*PPI-LRT*). When some parental genotypes are missing, one can use the expectation-maximization algorithm to fit the combined model. We also develop a second composite test (*PPI-CT*) based on a linear combination of the parent-phenotype-based test statistic and that from the traditional log-linear, transmission-based test. We evaluate the proposed methods through non-centrality parameter calculations and simulation studies and compare them to the previously proposed approaches, *parenTDT* and *combTDT*. We show that incorporation of parental phenotype data often improves statistical power. As illustration, we apply our method to a study of young-onset breast cancer and find that it improved precision for SNPs in *FGFR2* and that estimated relative risks based on triads are closely replicated using the parental data.

## Introduction

In the case-parents design, a design commonly used in genetic studies, investigators collect genotype (and exposure) data from affected individuals and their biological parents. They then carry out analyses designed to detect the distortions in transmission to affected offspring that are seen for SNPs associated with susceptibility. Such analyses typically neglect information on parental phenotype. That information may, however, be able to contribute additional power, especially for diseases that are not rare in a lifetime.

Purcell et al. (2005) developed ways to use parental phenotype information based on extending a within-sibship association model for quantitative traits (Fulker et al., 1999). A related method, *parenTDT*, is implemented in PLINK (Purcell et al., 2007). It is based on case-parent triads and exclusively captures information from parental phenotypes. The parent-phenotype information is combined with transmission-based information in the *combTDT*. Both *parenTDT* and *combTDT* have some drawbacks, however: they cannot use data from triads with missing genotypes and do not provide relative risk estimates. To address those issues, we develop alternative methods for exploiting parent-phenotype information in a case-parents design.

Family-based studies typically collect parental phenotype data because investigators want to ascertain first-degree family history. An example is the Two Sister Study (http://sisterstudy.niehs.nih.gov/English/2sis.htm), a family-based study of breast cancer diagnosed before age 50. DNA samples from the cases, their parents, when available, and unaffected control sisters (when parents were missing) were genotyped using the Illumina HumanOmniExpressExome-8v1 array. About 20% of the mothers had themselves been diagnosed with breast cancer. This study motivated us to develop our method and will serve to illustrate its application.

We first consider diseases that affect only one sex, e.g., female breast cancer or prostate cancer. Later we expand our method to permit study of diseases that affect both males and females.

## Methods

### Joint model for phenotypes in parents and offspring

Assuming a female-specific disease, let M, F, C denote the numbers of copies of the variant allele carried by the mother, father and daughter, respectively and let *D_M_* and *D_C_* denote the occurrence of the disease in the mother and daughter, respectively. The joint distribution of the parental phenotypes and triad genotypes can be factored into two parts as follows:

(1) $$\mathrm{Pr}\left(D_{M},M,F,C|D_{C}\right)=\mathrm{Pr}\left(M,F,C|D_{C}\right)\mathrm{Pr}\left(D_{M}|M,F,C,D_{C}\right)$$

Here the first factor contains the classical transmission-based information while the second factor contains information contributed by the parental phenotypes. These two sources of information can be modeled separately, as will be described below. Alternatively, the joint likelihood (1) can be maximized. The model for risk can be parameterized to impose equality of parental and offspring relative risks or to test that equality. The maximization can make use of the expectation-maximization (EM) algorithm when some parental genotypes are missing, provided that missingness is noninformative, that is, unrelated to the unmeasured genotype, conditional on the observed data (Dempster et al., 1977).

### Model for the transmission-based test

The transmission-based tests are based on modeling the distribution of triad genotypes conditional on the offspring's being affected. We postulate a robust log-linear risk model (Weinberg et al., 1998) as follows:

(2) $$\begin{array}{l} \text{ln}\left[\mathrm{Pr}(M,F,C|D_{C})\right]=\mu_{\left(M,F\right)}+\gamma_{1}I_{\left(C=1\right)}+\gamma_{2}I_{\left(C=2\right)}\\ \;+\ln(2)I_{\left(M=1,F=1,C=1\right)} \end{array}$$

Here the μ_(*M,F*)_ represent six mating-type parameters corresponding to the unordered parental genotypes under assumed mating symmetry for the allele under study. Here, mating symmetry means that in the source population Pr [*M* = *m, F* = *f*] = Pr [*M* = *f, F* = *m*], which implies that μ_(*M,F*)_ = μ_(*F,M*)_. These mating type parameters ensure that inferences about the γ_*j*_ use only information from transmissions, thereby conferring robustness against population stratification. Exponentiating γ_1_ and γ_2_ yields the relative risk for a child who carries 1 or 2 copies compared to one who carries no copies, i.e., *R*_1_ and *R*_2_. One can test for genetic effects of the variant by testing the null hypothesis that γ_1_ = γ_2_ = 0. The six mating type parameters confer robustness against population stratification by effectively forcing conditioning on the unordered parental genotypes at the studied locus. The co-dominant risk parameterization in (2) can be modified to accommodate a log-additive (as used in our simulations), dominant or recessive mode of inheritance.

### Modeling risk in a parent

We model the phenotype of the mother of an affected daughter using a log-binomial model as follows:

(3) $$\text{ln}\left[\mathrm{Pr}\left(D_{M}|M,F,D_{C}\right)\right]=\alpha_{\left(M,F\right)}+\beta_{1}I_{\left(M=1\right)}+\beta_{2}I_{\left(M=2\right)}$$

Here, the α_(*M,F*)_ represent six mating-type parameters corresponding to the unordered parental genotypes for the allele under study. Exponentiating β_1_ and β_2_ yields the relative risks for disease in the mothers who carry 1 or 2 copies compared to those who carry no copies, i.e., *R*_*m*1_ and *R*_*m*2_. One can test for genetic effects of the variant by testing the null hypothesis that β_1_ = β_2_ = 0.

In model (3), risk in mothers of cases depends on paternal genotype through the α_(*M,F*)_. These parameters provide proxy adjustment for sub-population membership; their inclusion confers resistance to bias due to genetic population stratification. Formal justification of robustness appears in the Supplementary Material. As explained there, the α_(*M,F*)_ allow baseline disease risks to differ across subpopulations; but control of stratification bias requires certain assumptions such as mating symmetry. Note also that the only informative pairs of parents (those who contribute to estimation of the β_*j*_) are parents who differ in their genotypes. Thus, robustness comes at a price. Also, we are assuming that the relative risks do not depend on epistasis with other causative variants that are enriched in parents of cases. That is, we assume that the conditioning on *D_C_* can be ignored in the modeling. (This assumption would certainly hold under the null so has no effect on validity of testing). The risk parameterization in (3) also can be modified to accommodate log-additive, dominant or recessive models. Model (3) can be fitted using procedures for generalized linear models that are readily available in statistical packages.

### Maternally-mediated genetic effects

The mother's genome governs the prenatal environment and consequently can be important to the development of the fetus, regardless of which alleles the mother transmitted. This effect of the mother's genome on the phenotype of her offspring (perhaps even long after birth) is called a maternally-mediated genetic effect. Suppose a fetus born to a mother with one or two copies of a risk allele has a relative risk of *S*_1_ or *S*_2_ for developing the disease compared to a fetus born to a mother with no copies. Model (2) above can be modified to accommodate maternally-mediated effects by introducing two maternal genetic-effect risk parameters, ρ_1_ and ρ_2_, as in Wilcox et al. (1998):

(4) $$\begin{array}{l} \text{ln}\left[\mathrm{Pr}(M,F,C|D_{C})\right]=\mu_{\left(M,F\right)}+\gamma_{1}I_{\left(C=1\right)}+\gamma_{2}I_{\left(C=2\right)}\\ \;+\;\rho_{1}I_{\left(M=1\right)}+\rho_{2}I_{\left(M=2\right)}\\ \;+\text{ ln}(2)I_{\left(M=1,F=1,C=1\right)} \end{array}$$

Exponentiating ρ_1_ and ρ_2_ yields the relative risk parameters *S*_1_ and *S*_2_. One caution is that, for model (4) to produce unbiased estimates of maternal effects there must not be an effect of the phenotype on reproduction that is differential for males vs. females. The parent-phenotype-based model (3) is not readily generalized to allow the study of maternally-mediated genetic effects unless one also has genotype data for the maternal grandmothers. In fact, when maternally-mediated genetic effects are present, the risk parameters in model (3) for mothers themselves are an amalgamation of effects from the alleles the mother inherited from her parents and maternally-mediated genetic effects arising from her mother's genotype.

### Combining information from the two generations

Let us assume from here on that model (4) has been applied to the case-parent data and provided no evidence for any maternally-mediated effects of the variant under study. We presume no maternal effects and turn next to the problem of devising a two-generational approach to studying the direct effects of the inherited variant. Using the likelihood in (1) together with models (2) and (3), one can base estimation and testing on information from both generations. When the data are complete and distinct risk parameters are used for offspring and parents, maximizing the combined likelihood confers no advantages—statistical results for complete data would be the same as with separate fits to models (2) and (3). When parental genotypes are missing or when a functional relationship between offspring and parent risk parameters is specified, however, using the combined model is beneficial. For example, one might specify that *R*_1_ = *R*_*m*1_ and *R*_2_ = *R*_*m*2_ to allow both offspring and parent information to contribute to risk estimation and testing. The relative risk parameters for mothers (*R*_*m*1_ and *R*_*m*2_) may not be identical to their counterparts for offspring (*R*_1_ and *R*_2_, respectively), in part because ages at onset may be systematically different, but setting them equal for modeling and testing may still be useful. We designate the resulting parent-phenotype-informed likelihood ratio test as *PPI-LRT*.

Alternatively, in the absence of missing genotypes, one can fit the transmission model and the parent-phenotype model separately and then combine the independent test statistics as a way to take advantage of the information provided by both generations. First, fit the same one-parameter risk model separately for the transmission model and for the parent-phenotype model (we use the log-additive parameterization though any one-parameter version could be used, such as a dominant model). Second, form a composite statistic as a linear combination of the test statistics from the separate fits. Let *X_t_* and *X_p_* be the one degree-of-freedom likelihood ratio test (LRT) chi-squared statistics where the subscripts, respectively, index “transmission” and “parental-phenotype.” Let σ_*t*_ and σ_*p*_ be the estimated standard errors of the estimated risk coefficients in the two models and let *S_t_* and *S_p_* be the signs of the corresponding coefficients. The composite statistic will take advantage of the fact that under alternatives to the null the relative risk in the parental phenotype model would likely be in the same direction as that in the transmission-based model. The composite statistic exploits that directional agreement to enhance power as follows:

$$Z_{C}=\frac{S_{p}\sqrt{X_{p}/\sigma_{p}^{2}}+S_{t}\sqrt{X_{t}/\sigma_{t}^{2}}}{\sqrt{1/\sigma_{p}^{2}+1/\sigma_{t}^{2}}}$$

*Z_C_* is a linear combination of signed versions of the square roots of one-degree-of-freedom chi-squared statistics. Under the null, each of these has a standard normal distribution so the linear combination also has a normal distribution, and the coefficients are chosen so the linear combination has variance 1. Consequently, under the null, *Z_C_* follows a standard normal distribution asymptotically and its square follows a chi-squared distribution with 1 degree of freedom. We call the test based on this statistic the parent-phenotype-informed composite test (*PPI-CT*).

### Diseases affecting both males and females

We next extend model (3) to accommodate diseases that can affect both males and females. If, conditional on mating type, the occurrence of disease in the mother is independent of the occurrence of disease in the father, we write the log-binomial model for parental disease status as:

(5) $$\begin{array}{l} \text{ln}\left[\mathrm{Pr}\left(\text{Disease in a particular parent}|M,F,D_{C}\right)\right]\\ =\alpha_{\left(M,F\right)}+\;\delta_{\left(M,F,\text{ parent is father}\right)}\\ \;+\beta_{1}I_{\left(M=1,\text{ parent is mother}\right)}+\beta_{2}I_{\left(M=2,\text{ parent is mother}\right)}\\ \;+\theta_{1}I_{\left(F=1,\text{ parent is father}\right)}+\theta_{2}I_{\left(F=2,\text{ parent is father}\right)} \end{array}$$

Exponentiating θ_1_ and θ_2_ yields estimates of the relative risks for fathers who carry 1 or 2 copies compared to those who do not carry the variant allele. The δ_(*M,F*, parent is father_) parameters correspond to adjustments to the α_(*M,F*)_ mating type parameters that are needed to accommodate possible differences between the baseline risks in fathers and those in mothers. This model also allows formal testing of the equality of the relative risks in mothers vs. fathers. When the relative risks do not appear to be different, one can fit a single set of relative risk parameters for mothers and fathers. Additionally, this model can be simplified by using log-additive, recessive or dominant coding.

If there is concern about possible population stratification such stratification could induce correlation between the parental phenotypes. Shared risk factors, e.g., related to diet, could also induce phenotype correlation in the two parents. This possible correlation could be explored by including, for example, the maternal phenotype as a predictor in the model for the paternal phenotype. Such correlation may necessitate the use of dependent data methods such as generalized estimating equations (GEE).

### *TDT* and *parenTDT*

The *TDT* (Spielman et al., 1993) is a widely used transmission-based test for di-allelic autosomal SNPs. The *TDT* has been extended to incorporate parental phenotype data, *parenTDT* (Purcell et al., 2007). The *parenTDT*, or the parental discordance test, which is a simplified version of the method proposed by Purcell et al. (2005), compares the number of alleles in affected vs. unaffected parents within each family. Due to the within-family matching, the *parenTDT* assumes only homogeneity within families rather than among families and is resistant to bias from population stratification. It discards as uninformative families where both parents are affected.

One can combine information from *TDT* and *parentTDT* into *combTDT* by summing counts of allele transmissions and allele counts of phenotypically-discordant parents (Purcell et al., 2007). Summing permits construction of a single chi-squared statistic for testing.

### Simulations and noncentrality parameter calculations

To evaluate power, we computed the noncentrality parameter (NCP) of a chi-square test statistic by applying a well-known method (Agresti, 1990), based on maximizing the likelihood using the expected counts under a specified alternative scenario as pseudo-data. The NCP for the likelihood ratio test is calculated as the change in deviance (twice the maximized log likelihood) between models that do vs. do not include the parameter(s) of interest. We calculated the NCPs for the TDT methods by similarly substituting expected counts for data in the test statistics. Power was then calculated from the NCP based on looking up the tail probabilities of the corresponding noncentral chi-squared distribution. For comparisons to TDT methods that provide one-degree-of-freedom (df) tests, we specified log-additive risks and constrained the relative risks for the parent's and offspring's phenotype to be the same so that our likelihood ratio tests also had one df. We used simulations to confirm Type I error rates and certain NCP-based power approximations and to investigate estimation. For each scenario we simulated and analyzed 5000 data sets. For the null scenarios, each simulated study consisted of 300, 600, or 1200 case-parents triads. For the alternative scenarios, each study consisted of 300 case-parent triads.

NCPs permit easy extrapolation of power results to any other sample size. For example, to calculate power for a different sample size, say *N*, instead of the 300 that we used, one can multiply the NCPs by *N*/300 to derive the new NCP and look up the corresponding tail probabilities for a noncentral chi-squared distribution.

To calculate expected counts, we first ignored mother's phenotype and calculated case-parent triad frequencies based on the specified allele frequency, population structure, and risk parameters [child inherited genetic effects (*R*_1_, *R*_2_) and maternally-mediated genetic effects (*S*_1_, *S*_2_)]. For homogenous populations, we assumed Hardy-Weinberg equilibrium (HWE); for structured populations, we admixed two homogenous populations with different allele frequencies and baseline risks. We then calculated the frequencies of triads with an affected mother by multiplying each triad frequency by the baseline risk in mothers of affected offspring (that is, the risk in non-carrier mothers, denoted maternal baseline risk, *MBR*), modified as needed according to the mother's genotype and the scenario's maternal relative risk parameters (*R*_*m*1_, *R*_*m*2_). We obtained the frequency distribution of triads with unaffected mothers by subtracting the mother-affected triad frequencies from the overall triad frequencies. In scenarios where both parents can be affected, we first calculated the triad genotype frequencies stratified on the mother's phenotype. We then considered the father's phenotype in a similar way to further stratify the frequencies. The expected counts were eventually obtained by multiplying the calculated frequencies by 300, the total number of triads in the study.

We used simulations to explore the Type I error rates under the null, with 5000 replicates of each simulated scenario. We considered null scenarios in a homogenous population with or without maternally-mediated genetic effects (*S*_1_ = *S*_2_ = 1; or *S*_1_ = 1.4 and *S*_2_ = *S*^2^_1_ = 1.96). We also considered several scenarios in which we assumed a stratified population with two equal-sized subpopulations where the ratio of baseline disease risks (risk in noncarriers) was either 1 or 3. The baseline disease risks in mothers of affected offspring in the two subpopulations were either the same (both equal to 0.2) or different (0.1 and 0.2 for the two subpopulations). The allele frequencies in the two subpopulations were either the same (both equal to 0.3) or different (0.1 and 0.3).

To evaluate power, we considered homogeneous populations where *R*_1_ = *R*_*m*1_ = 1.4 *R*_2_ = *R*_*m*2_ = 1.96 and *S*_1_ = *S*_2_ = 1. We first set the *MBR* as 0.2 and examined NCPs as a function of allele frequencies. We also studied power as a function of *MBR* under the same scenarios described above but with allele frequency set at 0.3.

We studied missing-genotype scenarios that were similar to the scenarios already mentioned except that 20% of the 300 triads had missing genotypes for fathers and the EM algorithm was used to maximize the likelihoods. The *MBR* was 0.2 and the allele frequencies ranged from 0.1 to 0.9. We also studied NCPs as a function of fraction of fathers missing while setting the allele frequency to 0.3.

Finally, we considered scenarios where either parent can be affected. We examined NCPs as a function of allele frequency while fixing the baseline risks in the mothers and fathers of affected offspring as 0.2 and 0.1, respectively.

For complete triads the parent-phenotype test can serve as a replication for triad-based findings, because it is statistically independent from the transmission-based test. We explore this potential use by comparing the relative risk estimates and z-statistics from the triad-based and parent-based analyses using simulations.

## Results

The information from the two generations can be combined in two ways: (1) the composite test based on *Z_C_* (*PPI-CT*); (2) the likelihood ratio test using the combined likelihood of formula (1) with log-additive mode of inheritance (*PPI-LRT*). Under log-additive risk scenarios these two tests perform very similarly (data not shown). That similarity might be expected: the *PPI-CT* is based on separate maximization of the transmission and parental-phenotype models, each with its own risk parameter, whereas the *PPI-LRT* maximizes a likelihood that is the product of the same two likelihoods but with the relative risks for mothers and for offspring constrained to be the same. Whether fit separately or together under the constraint, if the models are correctly-specified, the two tests should be similar for large samples. In the following comparisons, we used *PPI-CT* for scenarios with no missing data and *PPI-LRT* when some genotypes are missing.

For Type I error rates, we evaluated the following tests: the log-linear model with log-additive mode of inheritance for testing transmission distortion (*Offspring*), the log-binomial model with log-additive mode of inheritance for testing association with parental phenotypes (*Parent*), the composite test based on *Z_C_* (*PPI-CT*), *TDT, parenTDT*, and the combined TDT (*combTDT*). In the absence of maternally-mediated genetic effects and when the sample size was 300, *Parent* showed slightly inflated Type I error rates of 0.06 and 0.066 for scenarios 3 and 4. This inflation was due to the relatively small number of families that were both genetically informative and had an affected mother, which reduced the faithfulness of the asymptotic approximation. When the sample size was increased to 600 or 1200, all methods maintained nominal Type I error rates and *Offspring* and *Parent* provided correct *R*_1_ and *R*_*m*1_ estimates, respectively (Tables 1B,C). As expected, the *parenTDT* and *combTDT* tests showed inflated Type I error rates in the presence of maternally-mediated genetic effects (row 5 in Tables 1A–C). The Type I error rate of the proposed methods depends on the risk parameters *R*_*m*1_ and *R*_*m*2_. The *R_m_s* can sometimes represent an amalgamation of effects of the inherited genotype and maternally-mediated genetic effects (*R*_1_ and *R*_2_, *S*_1_ and *S*_2_, respectively): in that event one would expect the *R_m_s* to be between 1 and the maternally-mediated relative risk. For example, in row 5 the apparent relative risks for mother's genotype on her own phenotype (*R*_*m*1_ = 1.2, *R*_*m*2_ = 1.44) are between *R*_1_ = R_2_ = 1 and *S*_1_ = 1.4, *S*_2_ = 1.96, because her genotype is confounded by the causative genotype of her own mother in that one maternally-mediated effects scenario. The *Parent, PPI-CT, parenTDT*, and *combTDT* tests indeed show inflated Type I error rates when there are maternally-mediated effects.

**Table 1:** **Type I error rates under the null, with disease affecting one sex**.

**Simulation scenarios**					**Type I error rate**						**R_1_ (95% CI)**	
**MBR1, MBR2^a^**	**Fr1, Fr2^b^**	**BR1/BR2^c^**	***S*^d^_1_**	**R_m1_**	**Offspring**	**Parent**	**PPI-CT**	**TDT**	**parenTDT**	**combTDT**	**Offspring**	**Parent**
**(A) EACH DATA SET CONTAINS 300 FAMILIES**.												
0.2, 0.2	0.3, 0.3	1	1	1	0.046	0.054	0.047	0.046	0.049	0.048	1.00 (1.00, 1.00)	1.01 (1.00, 1.01)
0.2, 0.2	0.1, 0.3	3	1	1	0.054	0.056	0.051	0.053	0.048	0.048	1.00 (0.99, 1.00)	1.00 (0.99, 1.01)
0.1, 0.2	0.1, 0.3	1	1	1	0.045	0.060	0.050	0.045	0.052	0.048	1.00 (1.00, 1.01)	1.00 (0.99, 1.01)
0.1, 0.2	0.1, 0.3	3	1	1	0.046	0.066	0.049	0.046	0.055	0.047	1.00 (1.00, 1.01)	1.01 (0.99, 1.03)
0.2, 0.2	0.3, 0.3	1	1.4	1.2^e^	0.045	0.132	0.068	0.045	0.502	0.134	1.00 (1.00, 1.00)	1.21 (1.20, 1.22)
**(B) EACH DATA SET CONTAINS 600 FAMILIES**.												
0.2, 0.2	0.3, 0.3	1	1	1	0.049	0.05	0.047	0.049	0.047	0.046	1.00 (1.00, 1.00)	1.00 (0.99, 1.00)
0.2, 0.2	0.1, 0.3	3	1	1	0.055	0.051	0.046	0.055	0.047	0.049	1.00 (1.00, 1.00)	1.00 (0.99, 1.00)
0.1, 0.2	0.1, 0.3	1	1	1	0.046	0.053	0.047	0.045	0.046	0.047	1.00 (1.00, 1.00)	1.00 (0.99, 1.01)
0.1, 0.2	0.1, 0.3	3	1	1	0.052	0.057	0.052	0.052	0.054	0.048	1.00 (0.99, 1.00)	1.00 (0.99, 1.01)
0.2, 0.2	0.3, 0.3	1	1.4	1.2^e^	0.052	0.196	0.089	0.052	0.805	0.227	1.00 (1.00, 1.00)	1.20 (1.19, 1.21)
**(C) EACH DATA SET CONTAINS 1200 FAMILIES**.												
0.2, 0.2	0.3, 0.3	1	1	1	0.047	0.051	0.054	0.047	0.053	0.052	1.00 (1.00, 1.00)	1.00 (1.00, 1.00)
0.2, 0.2	0.1, 0.3	3	1	1	0.05	0.053	0.048	0.05	0.054	0.048	1.00 (1.00, 1.00)	1.00 (1.00, 1.00)
0.1, 0.2	0.1, 0.3	1	1	1	0.05	0.057	0.057	0.05	0.057	0.054	1.00 (1.00, 1.00)	1.00 (0.99, 1.00)
0.1, 0.2	0.1, 0.3	3	1	1	0.044	0.051	0.048	0.044	0.051	0.048	1.00 (1.00, 1.00)	1.00 (0.99, 1.01)
0.2, 0.2	0.3, 0.3	1	1.4	1.2^e^	0.05	0.323	0.141	0.05	0.98	0.411	1.00 (1.00, 1.00)	1.19 (1.19, 1.20)

a *Baseline disease risks in mothers of affected offspring in subpopulation 1 and 2*.

b *Allele frequencies in subpopulation 1 and 2*.

c *Ratio of baseline disease risk in subpopulation 1 vs. that in subpopulation 2*.

d *Relative risk for a child whose mother has one copy of the risk allele vs. those whose mother has none*.

e *A scenario without effects of the inherited genotype on offspring (R_1_ = R_2_ = 1) but with maternally-mediated genetic effects (S_1_ = 1.4, S_2_ = 1.96). The apparent relative risks for mother's genotype on her own phenotype are between the above two (R_m1_ = 1.2, R_m2_ = 1.44), because her genotype is confounded by the causative genotype of her own mother in this scenario*.

To illustrate that the robustness of the parental-phenotype-based test is due to the inclusion of the parental mating-type parameters, we omitted them from model (3) and fit a single intercept version to data from selected stratified scenarios. Type I error rates increased from 0.057 to 0.211 and from 0.051 to 0.183 in the two scenarios that we examined, documenting the need for mating-type-based intercept parameters.

**Figure 1:**
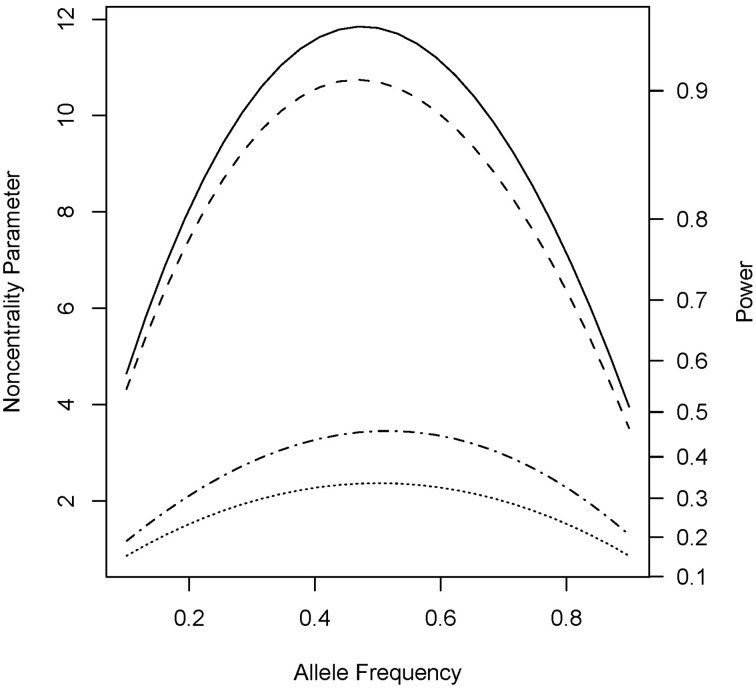
**Noncentrality parameter and power as a function of allele frequency**. All designs used 300 complete triads in a homogenous population under the risk scenario: *R*_1_ = *R*_*m*1_ = 1.4, *R*_2_ = *R*_*m*2_ = 1.96, and *S*_1_ = *S*_2_ = 1. The baseline risk in mothers of affected offspring is 0.2. Vertical axes: left, the chi-squared non-centrality parameter for a 1-df likelihood ratio test; right, power at α = 0.05. Horizontal axis shows the allele frequency ranging from 0.1 to 0.9. Curves: dot, *parenTDT*; dash-dot, *Parent*; dash, *combTDT*; solid, *PPI-CT*.

**Figure 2:**
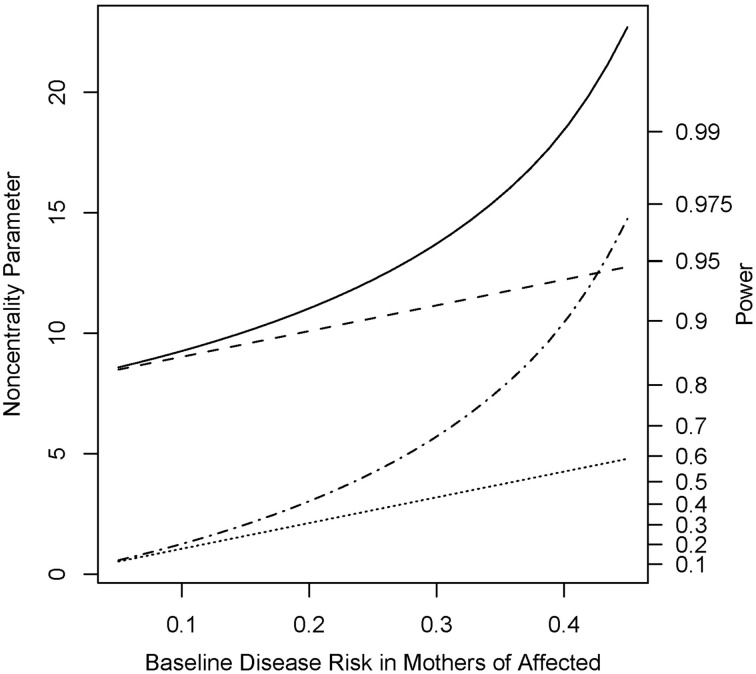
**Noncentrality parameter and power as a function of baseline risk in mothers of affected offspring**. All designs used 300 complete triads in a homogenous population under the risk scenario: *R*_1_ = *R*_*m*1_ = 1.4, *R*_2_ = *R*_*m*2_ = 1.96, and *S*_1_ = *S*_2_ = 1. The risk allele frequency is 0.3. Vertical axes: left, the chi-squared noncentrality parameter for a 1-df likelihood ratio test; right, power at α = 0.05. Horizontal axis shows the baseline risk in mothers of affected offspring ranging from 0.05 to 0.45. Curves: dot, *parenTDT*; dash-dot, *Parent*; dash, *combTDT*; solid, *PPI-CT*.

Under the alternative (non-null) scenarios, across different allele frequencies our proposed *Parent* test always performed better than *parenTDT*; so did our proposed *PPI-CT* vs. *combTDT* (Figure 1). NCP calculations were also validated by simulations and the *Parent* test showed unbiased relative risk estimates (Supplemental Table 1). As the baseline risk in the mothers of affected offspring increased from 0.05 to 0.45, the four tests all showed increased power. Again *Parent* and *PPI-CT* performed better than *parenTDT* and *combTDT* respectively, especially when the baseline risk was high (Figure 2 and Supplemental Figure 1).

**Figure 3:**
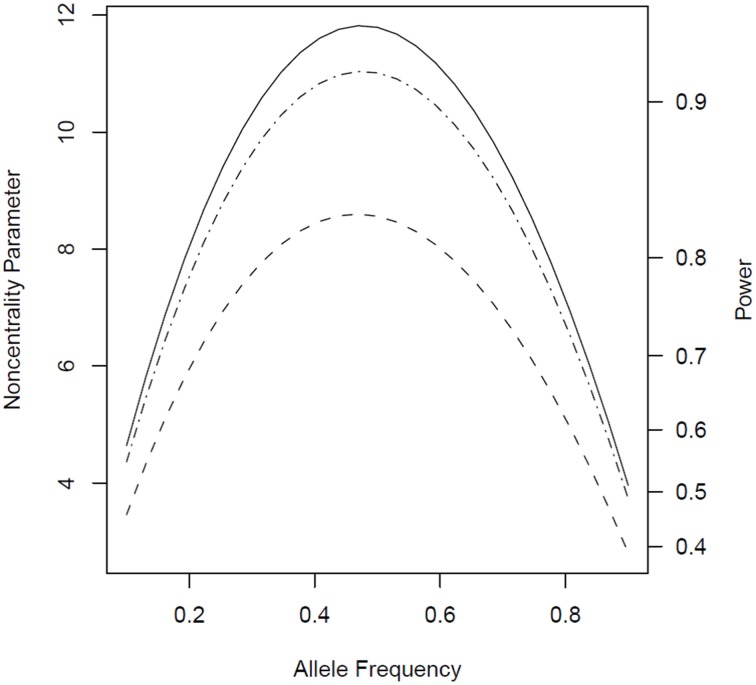
**Noncentrality parameter and power as a function of allele frequency in the presence of missing genotypes**. All designs used 300 triads from a homogeneous population with or without missing genotypes for 20% of the fathers. The risk scenario is: *R*_1_ = *R*_*m*1_ = 1.4, *R*_2_ = *R*_*m*2_ = 1.96, and *S*_1_ = *S*_2_ = 1. The baseline risk in mothers of affected offspring is 0.2. Vertical axes: left, the chi-squared noncentrality parameter for a 1-df likelihood ratio test; right, power at α = 0.05. Horizontal axis shows the allele frequency ranging from 0.1 to 0.9. Curves: dash, *combTDT* when genotypes are missing from 20% of the fathers; dash-dot, *PPI-LRT* when genotypes are missing from 20% of the fathers; solid, *PPI-LRT* test when there are no missing genotypes (this curve serves as a reference to show the impact of missing genotypes).

**Figure 4:**
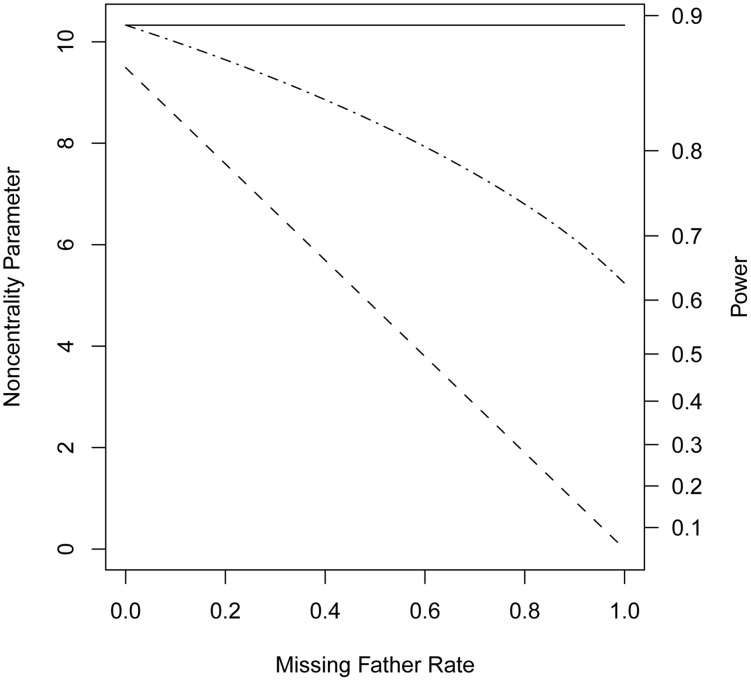
**Noncentrality parameter and power as a function of missing genotype rates**. All designs used 300 triads from a homogeneous population with a range of missing genotype rates in the fathers. The risk scenario is: *R*_1_ = *R*_*m*1_ = 1.4, *R*_2_ = *R*_*m*2_ = 1.96, and *S*_1_ = *S*_2_ = 1. The baseline risk in mothers of affected offspring is 0.2. Allele frequency was set at 0.3. Vertical axes: left, the chi-squared noncentrality parameter for a 1-df likelihood ratio test; right, power at α = 0.05. Horizontal axis shows the missing genotype rates in the fathers ranging from 0 to 1. Curves: dash, *combTDT*; dash-dot, *PPI-LRT*; solid, *PPI-LRT* test in the absence of missing genotypes which serves as a reference to show the impact of missing genotypes.

When genotypes were missing from 20% of the fathers, *PPI-LRT* was able to retrieve through use of the EM algorithm most of the lost power due to missing data. The TDT-based approach, however, suffered due to its need to discard families with missing genotypes (Figure 3). The *PPI-LRT*, as expected, did lose power as the missing rate increased (Figure 4). However, remarkably, even when all fathers' genotypes were missing the *PPI-LRT* still had reasonable power, while *combTDT* had no power at all (having no informative families left).

**Figure 5:**
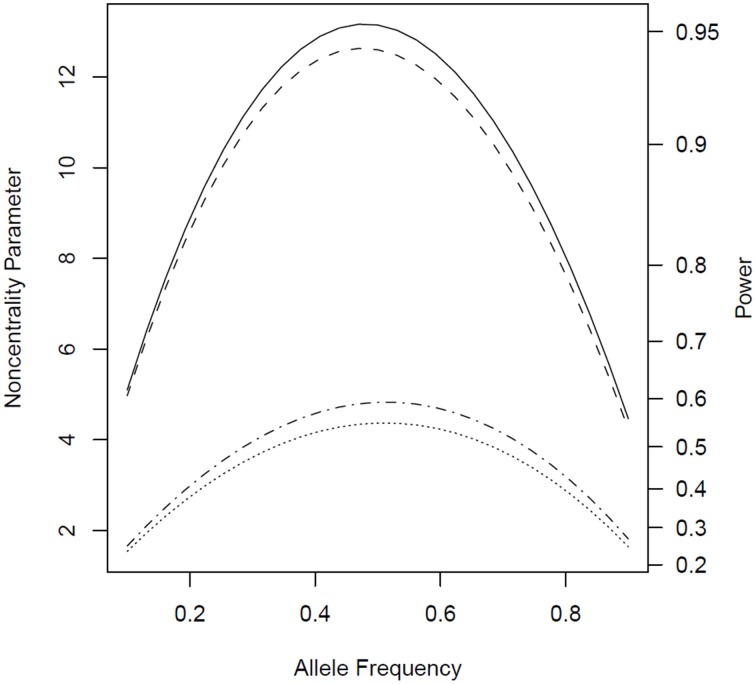
**Noncentrality parameter and power as a function of allele frequency under scenarios where both parents can be affected**. All designs used 300 complete triads in a homogenous population under the risk scenario: *R*_1_ = *R*_*m*1_ = 1.4, *R*_2_ = *R*_*m*2_ = 1.96, and *S*_1_ = *S*_2_ = 1. The baseline risk in mothers of affected offspring is 0.2 and 0.1 in fathers of affected offspring. Vertical axes: left, the chi-squared noncentrality parameter for a 1-df likelihood ratio test; right, power at α = 0.05. Horizontal axis shows the allele frequency ranging from 0.1 to 0.9. Curves: dot, *parenTDT*; dash-dot, *Parent*; dash, *combTDT*; solid, *PPI-CT*.

We observed similar patterns when the disease under consideration could affect both males and females (Figure 5). The power for the tests based on parental phenotypes and hence for the combined tests was higher compared to scenarios where only one sex was affected, due to the increased number of parent-affected families.

Another use of the parent-phenotype test when no genotypes are missing is as a replication for findings from the transmission-based test. We explored this role by examining the agreements in relative risk estimates and the corresponding *Z* statistics from the transmission-based and parent-phenotype-based tests under scenarios for Figure 1 (Supplemental Figure 2). Estimates from the two tests, although statistically independent, were almost always in the same direction, demonstrating that the test based on parental phenotype can serve to independently confirm transmission-based findings.

## Application

The fibroblast growth factor receptor 2 (*FGFR2*), a cell surface receptor with tyrosine-protein kinase activity, plays an essential role in the regulation of cell proliferation, differentiation, migration, and apoptosis. This gene has been consistently identified as a risk locus for breast cancer, and 7 SNPs in *FGFR2* have had a reported GWAS *p*-value less than 10^−5^ (Easton et al., 2007; Hunter et al., 2007; Gaudet et al., 2010; Elgazzar et al., 2012). We applied our method to genotypes of the 7 FGFR2 SNPs collected from 440 complete triads in the Two Sister Study (Table 2). For all 7 SNPs, the parent-phenotype test produced comparable relative risk estimates to the estimates based on the transmission model, demonstrating replicability across the two generations. With the added evidence from the parent-phenotype test, the combined tests showed smaller *p*-values. *Parent* and *PPI-CT* consistently gave smaller *p*-values than did *parenTDT* and *combTDT*, respectively.

**Table 2:** **Results from various tests applied to the breast cancer** ***FGFR2*** **example**.

**rs**	**Chr**	**Position**	**Test statistic *p*-value**						**Relative risk estimate**	
			**Offspring^a^**	**Parent^a^**	**PPI-CT^a^**	**TDT**	**parenTDT**	**combTDT**	**Daughter**	**Mother**
rs3750817	10	123332577	0.337	0.279	0.181	0.337	1.000	0.379	0.91 (0.74, 1.11)	0.80 (0.53, 1.20)
rs2981579	10	123337335	0.010	0.123	0.003	0.010	0.467	0.008	1.29 (1.06, 1.57)	1.41 (0.90, 2.21)
rs1078806	10	123338975	0.017	0.172	0.006	0.013	0.529	0.011	1.27 (1.04, 1.56)	1.38 (0.86, 2.19)
rs2981578	10	123340311	0.020	0.116	0.006	0.020	0.564	0.018	0.79 (0.65, 0.97)	0.72 (0.48, 1.08)
rs2981575	10	123346116	0.008	0.265	0.004	0.008	0.714	0.010	1.30 (1.07, 1.59)	1.29 (0.83, 2.00)
rs1219648	10	123346190	0.019	0.197	0.008	0.019	0.617	0.018	1.27 (1.04, 1.54)	1.35 (0.85, 2.12)
rs2981582	10	123352317	0.027	0.142	0.009	0.027	0.541	0.023	1.25 (1.03, 1.52)	1.40 (0.89, 2.20)

a *These tests are likelihood ratio tests based on a log-additive mode of inheritance*.

## Discussion

Several methods have been proposed to extract information from parental phenotypes. Whittemore and Tu (1998) proposed a founder statistic that can take advantage of parent phenotype information by comparing parent genotypes with those of the reference population weighted by the phenotype value. This founder statistic, however, requires correct specification of the reference-genotype probabilities and is susceptible to bias under population stratification. Purcell et al. (2005) proposed a method that incorporates the parent phenotypes by inclusion of parental genotype-phenotype correlation terms in the association tests. This method can be used for dichotomous traits through a liability-threshold-model approach. We use a simplified version of this method (*parenTDT*) as a comparison in this paper. Yu et al. (2013) proposed a method to incorporate parental information into family-based association tests but instead of using the information in direct testing of association, their method uses parental information to infer the mode of inheritance. A method that can make use of family-history data in population-based case-control association studies has also been proposed (Ghosh et al., 2014). Because parental genotypes are not available for direct evaluation in a case-control study, that method treats family-history as a “phenotype” and accounts for the expected attenuation in strength of the association. Evidence from the case-control comparison and family-history comparison is then combined using a meta-analysis approach.

The method we have proposed here is robust to population stratification, can provide relative risk estimates, and can handle missing genotypes through use of the EM algorithm. With complete triads, the parent-based test is independent of the transmission-based test and can serve as an internal replicate for findings based on transmission distortion. With incomplete triads, however, requiring used of the EM to maximize the likelihood, the independence of these two tests no longer holds.

Some caveats need to be mentioned. The log-binomial model will resist convergence (Williamson et al., 2013) when excursions during iteration take fitted probabilities below 0 or above 1.0. In analyzing our simulated data we avoided this issue by assigning reasonable starting values.

Another issue is that without grandparent genotype data the parent-based model cannot distinguish a maternally-mediated (here grandmother-mediated) effect from an inherited gene effect. Consequently, the estimated relative risks can be biased if a maternally-mediated effect is involved. These problems also apply to other approaches, such as a case-control association study, but they are rarely acknowledged. Finally, while incorporating information related to parental phenotypes should always add power, that power gain will be modest if the disease is rare in parents of affected offspring. Breast cancer was a likely candidate, because its lifetime risk is so high (approximately 12% per Howlader et al., 2012).

Some assumptions beyond the usual Mendelian inheritance assumption are needed for strict validity of the estimation of relative risks. Because *D_C_* is not incorporated as a predictor in model (3), epistasis can potentially produce bias (away from the null) in estimation based on that parental phenotype model if genetic background acts synergistically with the variant under analysis. However, there was little evidence of that phenomenon in our breast cancer example.

Family-based studies often collect parent phenotype data and consequently improved methods for using that data to enhance genetic risk assessment will be widely applicable. Our proposed strategy for combining parent-phenotype information with genotype data from case-parents designs resists bias due to population structure, delivers improved power, provides relative risk estimates and can handle missing genotypes, thereby offering advantages over existing approaches.

### Conflict of interest statement

The authors declare that the research was conducted in the absence of any commercial or financial relationships that could be construed as a potential conflict of interest.

## References

[B1] Agresti A. (1990). Categorical Data Analysis. New York, NY: John Wiley & Sons.

[B2] Dempster A. P. Laird N. M. Rubin D. B. (1977). Maximum Likelihood from Incomplete Data via the *EM* Algorithm. J. R. Stat. Soc. B Methodol. 39, 1–38.

[B3] Easton D. F. Pooley K. A. Dunning A. M. Pharoah P. D. Thompson D. Ballinger D. G.. (2007). Genome-wide association study identifies novel breast cancer susceptibility loci. Nature 447, 1087–1093. 10.1038/nature05887 17529967 PMC2714974

[B4] Elgazzar S. Zembutsu H. Takahashi A. Kubo M. Aki F. Hirata K.. (2012). A genome-wide association study identifies a genetic variant in the SIAH2 locus associated with hormonal receptor-positive breast cancer in Japanese. J. Hum. Genet. 57, 766–771. 10.1038/jhg.2012.108 22951594

[B5] Fulker D. W. Cherny S. S. Sham P. C. Hewitt J. K. (1999). Combined linkage and association sib-pair analysis for quantitative traits. Am. J. Hum. Genet. 64, 259–267. 10.1086/302193 9915965 PMC1377724

[B6] Gaudet M. M. Kirchhoff T. Green T. Vijai J. Korn J. M. Guiducci C.. (2010). Common genetic variants and modification of penetrance of BRCA2-associated breast cancer. PLoS Genet. 6:e1001183. 10.1371/journal.pgen.1001183 21060860 PMC2965747

[B7] Ghosh A. Hartge P. Kraft P. Joshi A. D. Ziegler R. G. Barrdahl M.. (2014). Leveraging family history in population-based case-control association studies. Genet. Epidemiol. 38, 114–122. 10.1002/gepi.21785 24408355 PMC6314034

[B8] Howlader N. Noone A. Krapcho M. Neyman N. Aminou R. Waldron W. (2012). SEER Cancer Statistics Review, 1975-2009 (Vintage 2009 Populations). Bethesda, MD: National Cancer Institute.

[B9] Hunter D. J. Kraft P. Jacobs K. B. Cox D. G. Yeager M. Hankinson S. E.. (2007). A genome-wide association study identifies alleles in *FGFR2* associated with risk of sporadic postmenopausal breast cancer. Nat. Genet. 39, 870–874. 10.1038/ng2075 17529973 PMC3493132

[B10] Purcell S. Neale B. Todd-Brown K. Thomas L. Ferreira M. A. Bender D.. (2007). PLINK: a tool set for whole-genome association and population-based linkage analyses. Am. J. Hum. Genet. 81, 559–575. 10.1086/519795 17701901 PMC1950838

[B11] Purcell S. Sham P. Daly M. J. (2005). Parental phenotypes in family-based association analysis. Am. J. Hum. Genet. 76, 249–259. 10.1086/427886 15614722 PMC1196370

[B12] Spielman R. S. McGinnis R. E. Ewens W. J. (1993). Transmission test for linkage disequilibrium: the insulin gene region and insulin-dependent diabetes mellitus (IDDM). Am. J. Hum. Genet. 52, 506–516. 8447318 PMC1682161

[B13] Weinberg C. R. Wilcox A. J. Lie R. T. (1998). A log-linear approach to case-parent-triad data: assessing effects of disease genes that act either directly or through maternal effects and that may be subject to parental imprinting. Am. J. Hum. Genet. 62, 969–978. 10.1086/301802 9529360 PMC1377041

[B14] Whittemore A. S. Tu I. P. (1998). Simple, robust linkage tests for affected sibs. Am. J. Hum. Genet. 62, 1228–1242. 10.1086/301820 9599188 PMC1377107

[B15] Wilcox A. J. Weinberg C. R. Lie R. T. (1998). Distinguishing the effects of maternal and offspring genes through studies of “case-parent triads.” Am. J. Epidemiol. 148, 893–901. 10.1093/oxfordjournals.aje.a009715 9801020

[B16] Williamson T. Eliasziw M. Fick G. H. (2013). Log-binomial models: exploring failed convergence. Emerg. Themes Epidemiol. 10:14. 10.1186/1742-7622-10-14 24330636 PMC3909339

[B17] Yu Z. Gillen D. Li C. F. Demetriou M. (2013). Incorporating parental information into family-based association tests. Biostatistics 14, 556–572. 10.1093/biostatistics/kxs048 23266418 PMC3732025

