# Supplementary material for: Using parental phenotypes in case-parent studies
Source: Front Genet. 2015 Jun 23;6:221. doi: 10.3389/fgene.2015.00221 (PMC4477179; doi:10.3389/fgene.2015.00221)
Supplement: Supplementary file 1 [file DataSheet1.DOCX]

Supplemental Table 1. Power and R1 estimates in a homogenous population. The baseline disease risk in the case mothers is 0.2. Relative risks are: *R_1_*=*R_m1_*=1.4, *R_2_*=*R_m2_*= 1.96 and *S_1_*=*S_2_*= 1.

| Allele Fr | Power | | | | | |  | R_1_ (95% CI) | |
| --- | --- | --- | --- | --- | --- | --- | --- | --- | --- |
|  | Offspring | Parent | *PPI-CT* | TDT | parenTDT | combTDT |  | Offspring | Parent |
| 0.1 | 0.48 | 0.20 | 0.57 | 0.48 | 0.15 | 0.56 |  | 1.42 (1.40,1.43) | 1.40 (1.37,1.43) |
| 0.2 | 0.67 | 0.31 | 0.79 | 0.67 | 0.23 | 0.77 |  | 1.40 (1.39,1.41) | 1.41 (1.39,1.44) |
| 0.3 | 0.79 | 0.42 | 0.90 | 0.79 | 0.32 | 0.89 |  | 1.41 (1.40,1.42) | 1.43 (1.41,1.45) |
| 0.4 | 0.83 | 0.42 | 0.92 | 0.83 | 0.32 | 0.90 |  | 1.41 (1.40,1.42) | 1.41 (1.40,1.43) |
| 0.5 | 0.81 | 0.45 | 0.93 | 0.81 | 0.35 | 0.92 |  | 1.40 (1.39,1.41) | 1.41 (1.40,1.43) |
| 0.6 | 0.80 | 0.43 | 0.90 | 0.80 | 0.31 | 0.88 |  | 1.40 (1.39,1.41) | 1.40 (1.39,1.42) |
| 0.7 | 0.74 | 0.42 | 0.87 | 0.74 | 0.31 | 0.84 |  | 1.40 (1.39,1.41) | 1.42 (1.41,1.44) |
| 0.8 | 0.62 | 0.33 | 0.75 | 0.62 | 0.22 | 0.73 |  | 1.41 (1.39,1.42) | 1.40 (1.38,1.42) |
| 0.9 | 0.36 | 0.22 | 0.51 | 0.36 | 0.15 | 0.45 |  | 1.40 (1.39,1.42) | 1.43 (1.40,1.46) |

Figure Legends:

Supplemental Figure 1. Power based on simulations. We considered a homogeneous population and simulated 1000 data set for each scenario with 300 complete triads in each data set. The relative risks are: *R_1_*=*R_m1_*=1.4, *R_2_*=*R_m2_*= 1.96 and *S_1_*=*S_2_*= 1 and the risk allele frequency is 0.3. Vertical axes shows the power. Horizontal axis shows the baseline risk in the mothers of affected ranging from 0.05 to 0.45. Curves are the fitted lines based on the observed powers (denoted by symbols): dot, parenTDT; dash-dot, log-binomial of parental phenotypes; dash, combTDT; solid, *PPI-CT* test of transmission-based test and log-binomial parent-phenotype test.

Supplemental Figure 2. Scatter plots of relative risk estimates and the corresponding z-statistics from the (independent) parent-phenotype based test and transmission-based test. All designs used 300 complete triads in a homogenous population under the risk scenario: *R_1_*=*R_m1_*=1.4, *R_2_*=*R_m2_*= 1.96 and *S_1_*=*S_2_*= 1. The baseline risk in the case mothers is 0.2. The allele frequency is 0.1 for a) and b); 0.2 for c) and d); 0.3 for e) and f); 0.4 for g) and h); 0.5 for i) and j); 0.6 for k) and l); 0.7 for m) and n); 0.8 for o) and p); 0.9 for q) and r).

Subplots a), c), e), g), i), k), m), o), q): Horizontal axis shows the z-score statistics from the transmission-based test. Vertical axis shows the z-score statistics from the tests based on parental phenotypes. The upper right quadrant (shaded) is the area where the z-statistics from the two tests are in the same and correct direction. The vertical and horizontal solid lines (at 1.96) in the upper quadrant show the cut-off for significant test at α=0.05 level. Hence, the upper right quadrant of the shaded area (the darkest grey) denotes the area where both tests reject the null at α=0.05 level. The normal density plots based on the means and variances of the vertical and horizontal axis values are shown on the corresponding margins.

Subplots b), d), f), h), j), l), n), p), r): Horizontal axis shows the beta coefficients from the transmission-based test. Vertical axis shows the beta coefficients from the tests based on parental phenotypes. The upper right quadrant (shaded) includes the simulations where the beta coefficients are in the same and correct direction. The normal density plots based on the means and variances of the vertical and horizontal axis values are shown on the corresponding margins. In the subplots, the vertical and horizontal solid lines show the observed mean coefficients and the dash lines show the true coefficient values. The statistical significance at α=0.05 level is denoted by symbols: open circle, neither test is significant; triangle, only transmission-based test is significant; cross, only test based on parental phenotypes is significant; filled circle, both tests are significant.

Supplemental Figure 1

Supplemental Figure 2


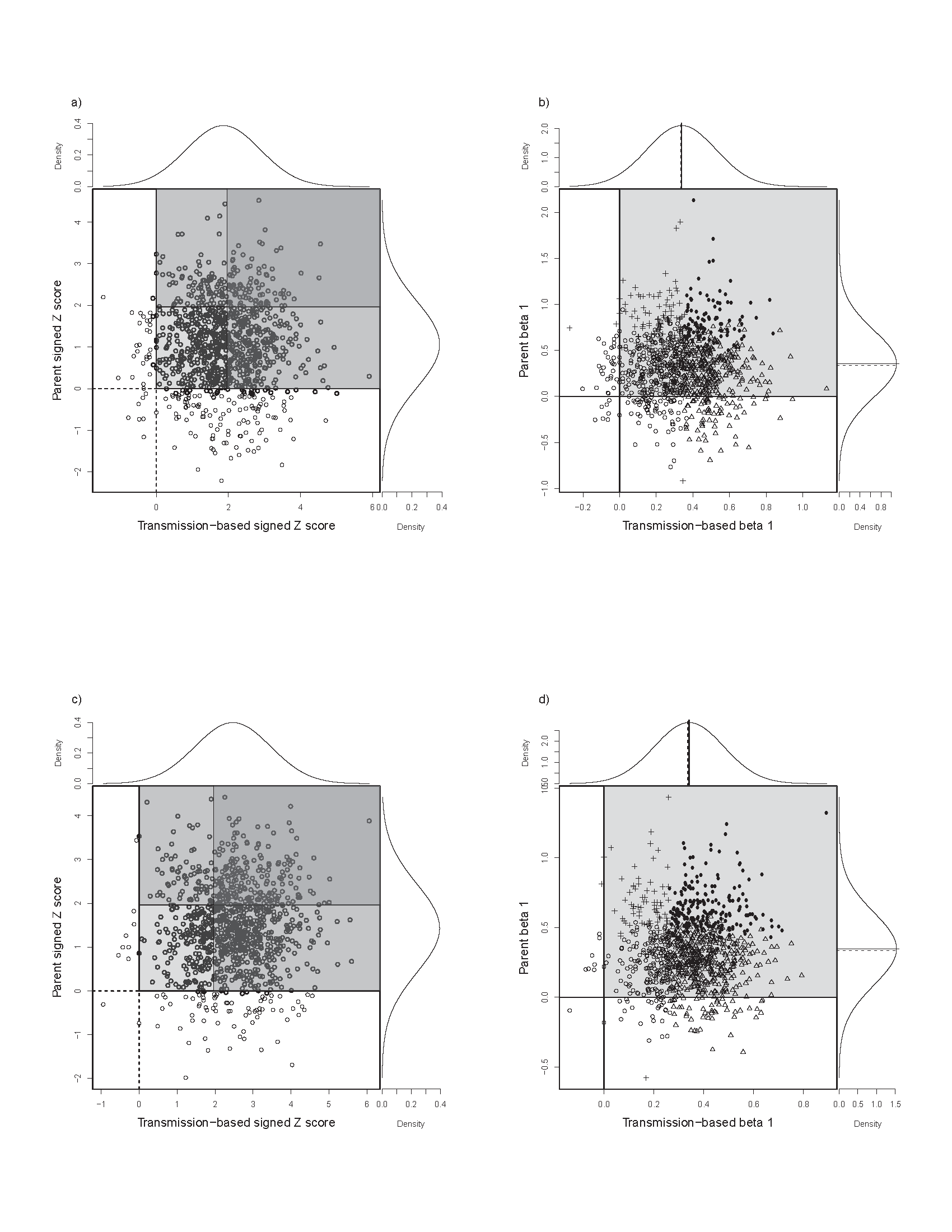


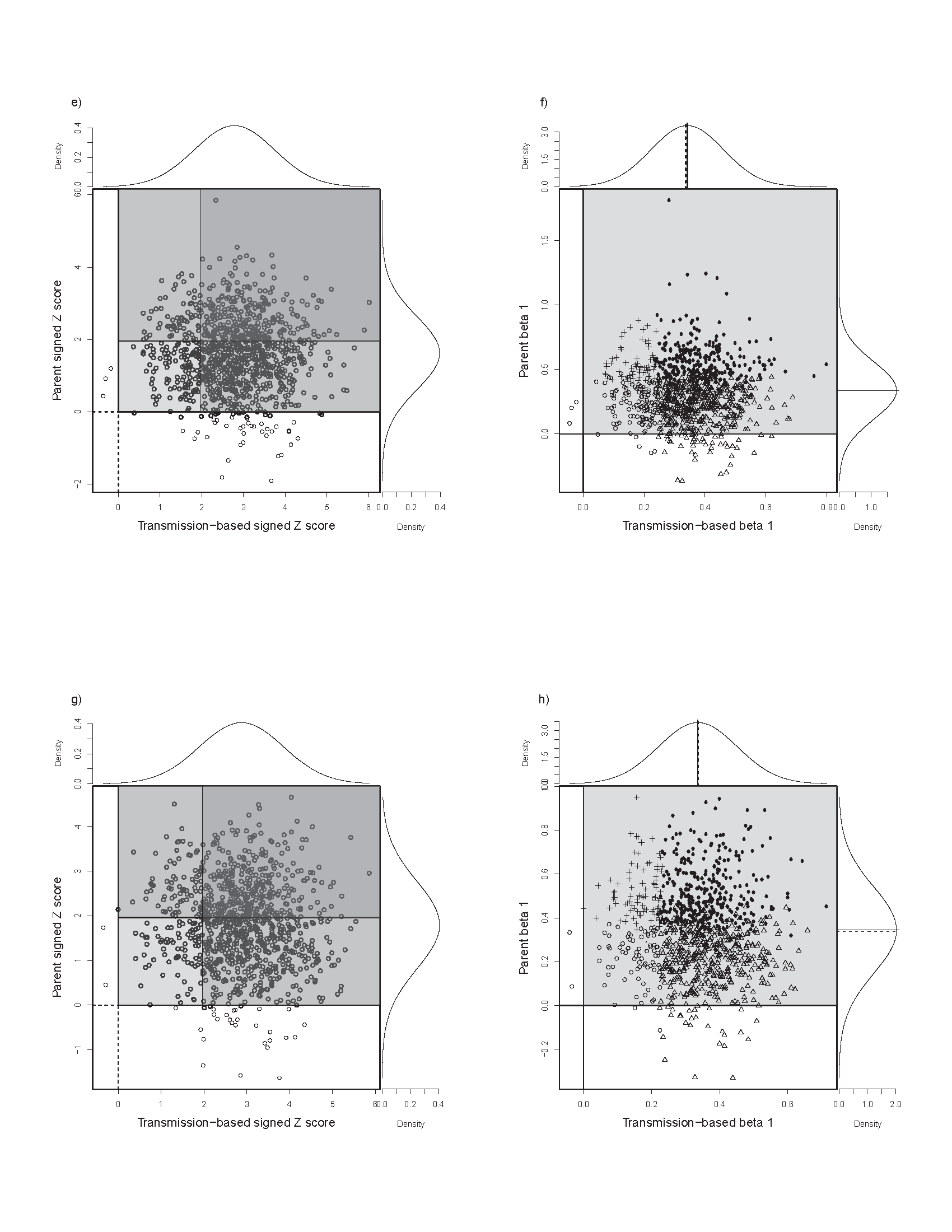


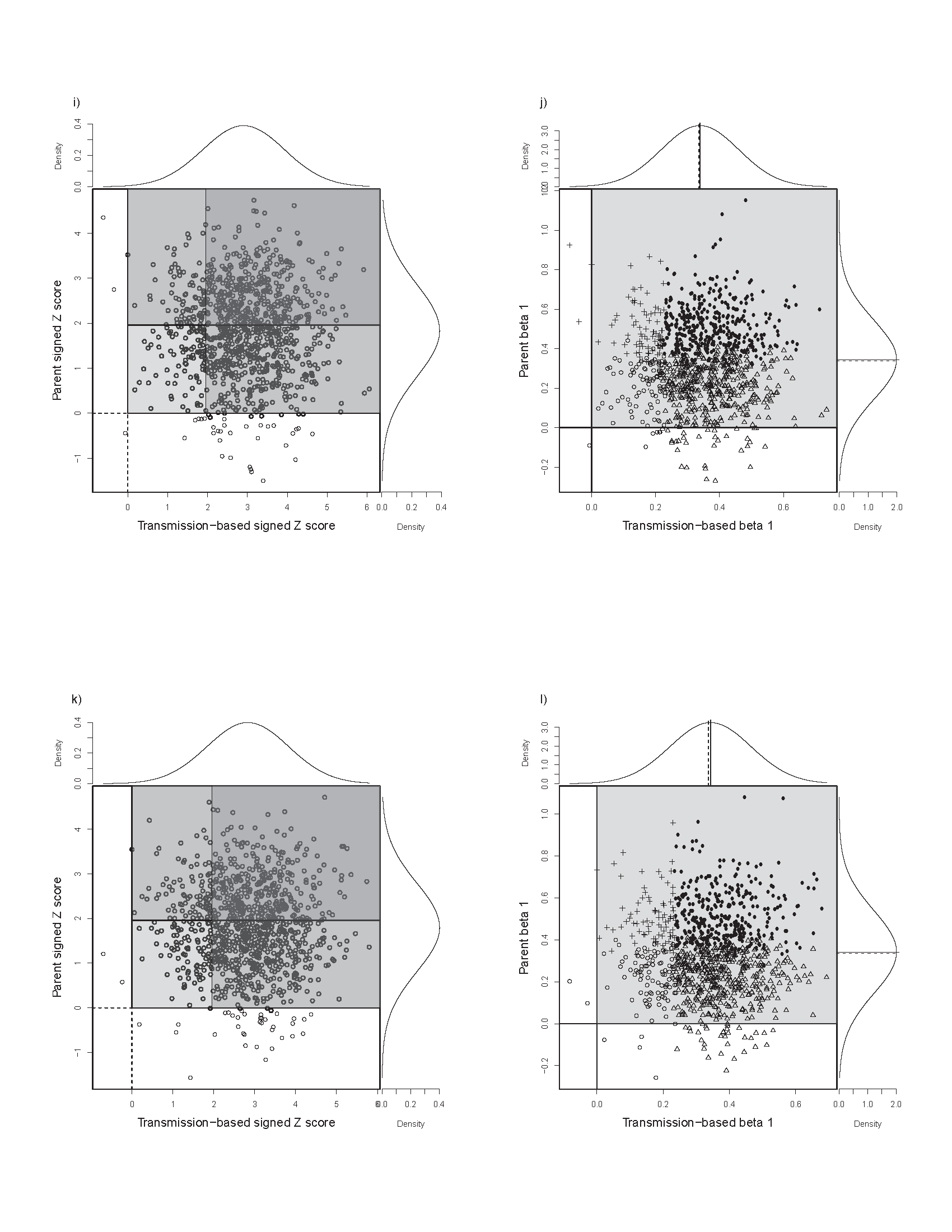


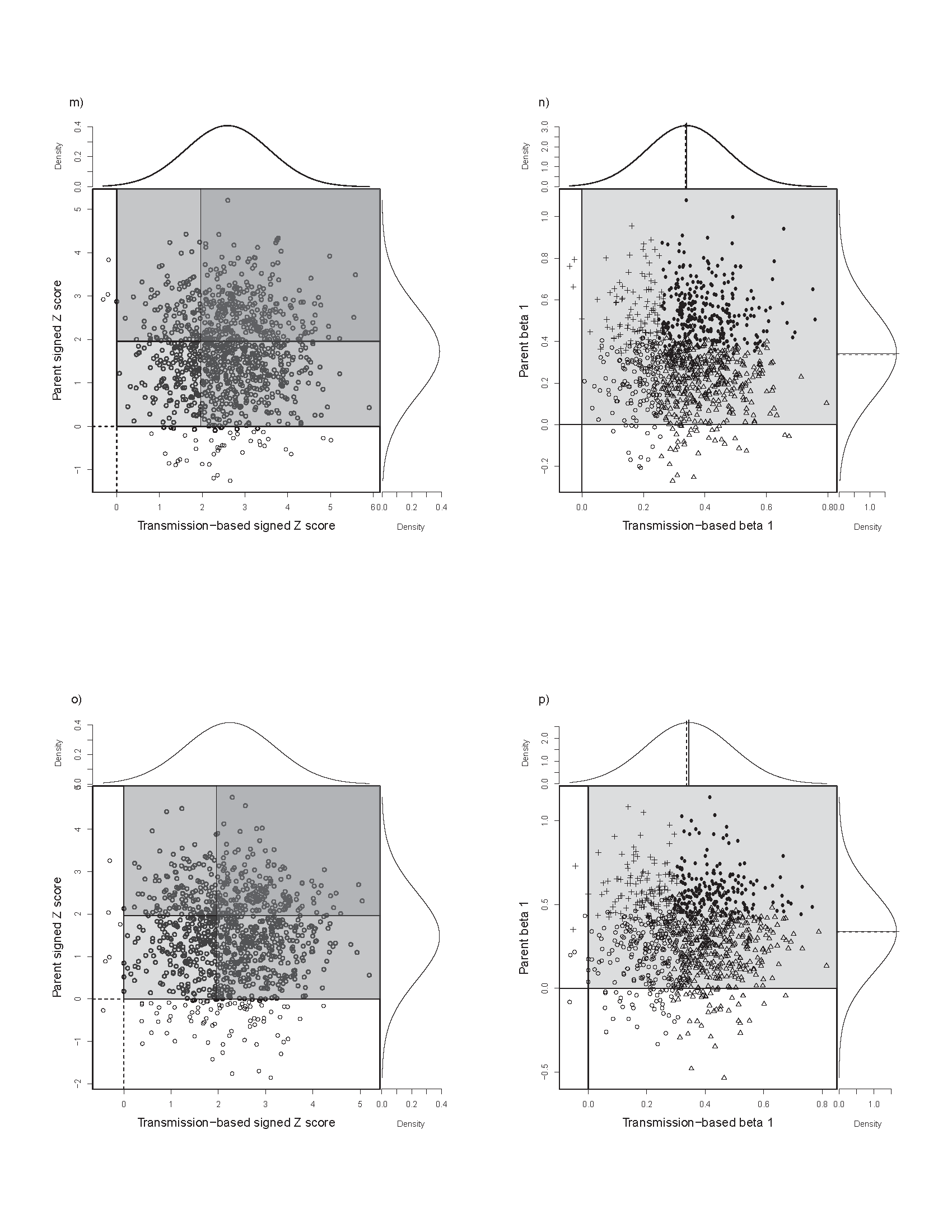


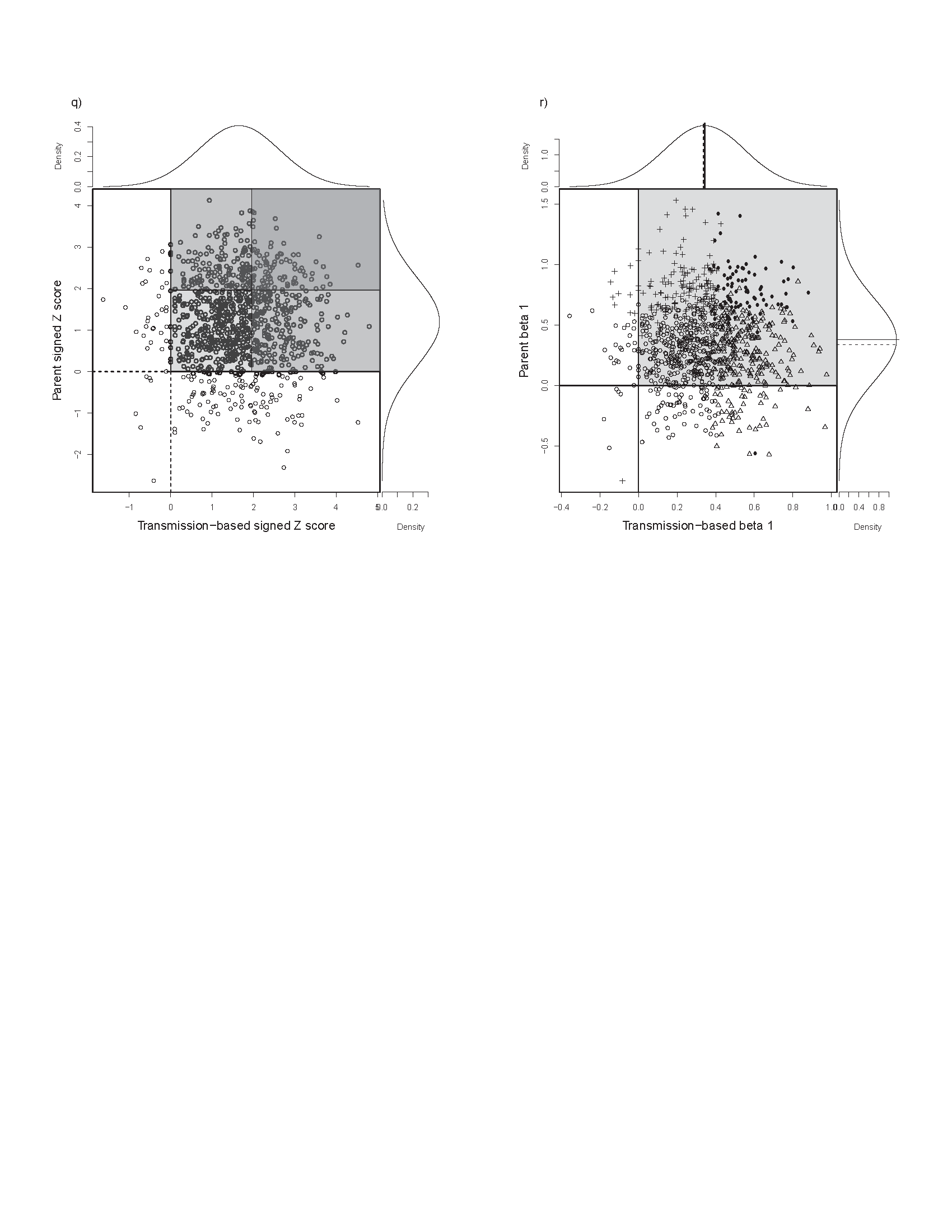


### Supplemental Proof. On robustness of the maternal risk model to population structure

To show that the maternal risk model in the manuscript, given as equation (3), enables inference that resists bias from population structure, we conceptualize a genetically-stratified population as a mix of undifferentiated (by the investigator) subpopulations of people who marry within their group, and postulate a model for maternal risk within each genetically homogeneous subpopulation. We then derive the population model by averaging over subpopulations.

Let $D_{M}$ be the event that the mother has the disease; let $S$ index the subpopulation, and assume that within each subpopulation, $s$, the relative risk for the mother depends on her genotype, $M=m$, but does not depend on the genotype of her child’s father, $F=f$. The baseline risk (risk among non-carriers) will be denoted $exp\left\{ \eta_{s} \right\}$, and can vary across subpopulations, as indicated by the subscript; however, we assume that the relative risks to the mother ($\beta_{1}$ for 1 copy and $\beta_{2}$ for 2 copies of the risk allele) are the same in all subpopulations. Thus, within each subpopulation, $s$:

$$\Pr\left[ D_{M} | M=m,S=s \right]=exp\left\{ \eta_{s}+\beta_{1}I_{\left( m=1 \right)}+\beta_{2}I_{\left( m=2 \right)} \right\}. (A1)$$

Because maternal relative risk does not depend on paternal genotype, the same model holds for each pair of maternal and paternal genotypes. Thus:

$$\Pr\left[ D_{M} | M=m,F=f, S=s \right]=exp\left\{ \eta_{s}+\beta_{1}I_{(m=1)}+\beta_{2}I_{(m=2)} \right\}. (A2)$$

Because the subpopulations are unobservable, we average across subpopulations, conditioning on $M \mathrm{and} F,$ to get a model based on observable quantities:

$$\Pr\left[ D_{M} | M=m,F=f \right]=\sum_{s} \Pr\left[ D_{M} | M=m,F=f, S=s \right]\Pr\left[ S=s | M=m,F=f \right]$$

$= exp\left\{ \beta_{1}I_{(m=1)}+\beta_{2}I_{(m=2)} \right\}\sum_{s} exp\left\{ \eta_{s} \right\}\Pr\left[ S=s | M=m,F=f \right]$

$= exp\left\{ \alpha_{mf}+\beta_{1}I_{(m=1)}+\beta_{2}I_{(m=2)} \right\}. (A3)$

The last step follows because, for each of the nine possible $(m,f)$-pairs, $\sum_{s} exp\left\{ \eta_{s} \right\}\Pr\left[ S=s | M=m,F=f \right]$is a weighted average of the $exp\left\{ \eta_{s} \right\}$ and is a function only of $m \mathrm{and} f$. We denote its value by $exp\left\{ \alpha_{mf} \right\}$. By including the $\alpha_{mf}$ parameters, model (A3) properly reflects the process of averaging across unobserved subpopulations and is, in that sense, correctly specified.

Model (A3) with nine mating-type parameters and two genetic risk parameters is over-parameterized, however, so the coefficients are not identifiable. To resolve the over-parameterization, we impose the restriction that $\alpha_{mf}=\alpha_{fm}$ for all $m\neq f$, and denote the resulting six mating-type parameters that depend on the unordered pair of parental genotypes by $\alpha_{(m,f)}$, as in model (3) of the paper. This restriction would hold, for example, when the weights are invariant under switching of the parental genotypes, *i.e.*, if $\Pr\left[ S=s | M=m,F=f \right]=\Pr\left[ S=s | M=f,F=m \right] for all m\neq f$.

Mating symmetry in every subpopulation is sufficient to meet this invariance criterion. Suppose that, for each $(m,f)$-pair and every $s$ in the population under study, $\Pr\left[ M=m,F=f | S=s \right]=\Pr\left[ M=f,F=m | S=s \right].$ Under that assumption, invariance under switching of parental genotypes follows from:

$$\Pr\left[ S=s | M=m,F=f \right]=\frac{\Pr\left[ M=m,F=f | S=s \right]\Pr\left[ S=s \right]}{\sum_{s} \Pr\left[ M=m,F=f | S=s \right]\Pr\left[ S=s \right]}$$

$$=\frac{\Pr\left[ M=f,F=m | S=s \right]\Pr\left[ S=s \right]}{\sum_{s} \Pr\left[ M=f,F=m | S=s \right]\Pr\left[ S=s \right]}=\Pr\left[ S=s | M=f,F=m \right].$$

Although more complex conditions also guarantee robustness, the assumption of mating symmetry in every subpopulation seems both plausible and intuitive, though difficult to verify.
